# Brachial Approach As an Alternative Technique of Fibrin Sheath Removal for Implanted Venous Access Devices

**DOI:** 10.3389/fsurg.2017.00020

**Published:** 2017-04-10

**Authors:** Charalampos Sotiriadis, Steven David Hajdu, Francesco Doenz, Salah D. Qanadli

**Affiliations:** ^1^Lausanne University Hospital, Lausanne, Switzerland

**Keywords:** brachial access, fibrin sheath removal, implanted venous access devices, vein, endovascular treatment

## Abstract

Implanted venous access device (IVAD) late dysfunction is commonly caused by fibrin sheath formation. The standard method of endovascular fibrin sheath removal is performed *via* the femoral vein. However, it is not always technically feasible and sometimes contraindicated. Moreover, approximately 4–6 h of bed rest is necessary after the procedure. In this article, we describe an alternative method of fibrin sheath removal using the brachial vein approach in a young woman receiving chemotherapy for breast cancer. The right basilic vein was punctured, and a long 6°F introducer sheath was advanced into the right subclavian vein. Endovascular maneuvers consisted on advancing Atrieve™ Vascular Snare 15–9 mm after catheter insertion in the superior vena cava through a 5.2°F Judkins left catheter. IVAD patency was restored without any complication, and the patient was discharged immediately after the procedure. In conclusion, fibrin sheath removal from an obstructed IVAD could be performed *via* the right brachial vein. Further research is necessary in order to prove efficacy of this technique.

## Introduction

The use of implanted venous access devices (IVADs) has significantly improved the quality of life in patients requiring long-term intravenous therapy (1). They are mainly used when repeated venous punctures are required for administrating chemotherapy or antibiotics. However, there are several complications concerning the use of these devices, classified as either early, when they appear immediately after placement or late, when they appear after 1 month (2). The most common late complication is partial catheter obstruction when blood aspiration is impossible *via* a functioning injection port (2). Fibrin sheath formation is frequently responsible for this phenomenon thus requiring removal using the internal snare technique to restore patency *via* a femoral venous access (1). Unfortunately, femoral access is not always available. The authors describe a case of fibrin sheath removal, which was successfully performed *via* brachial venous access.

## Case Presentation

**Figure 1:**
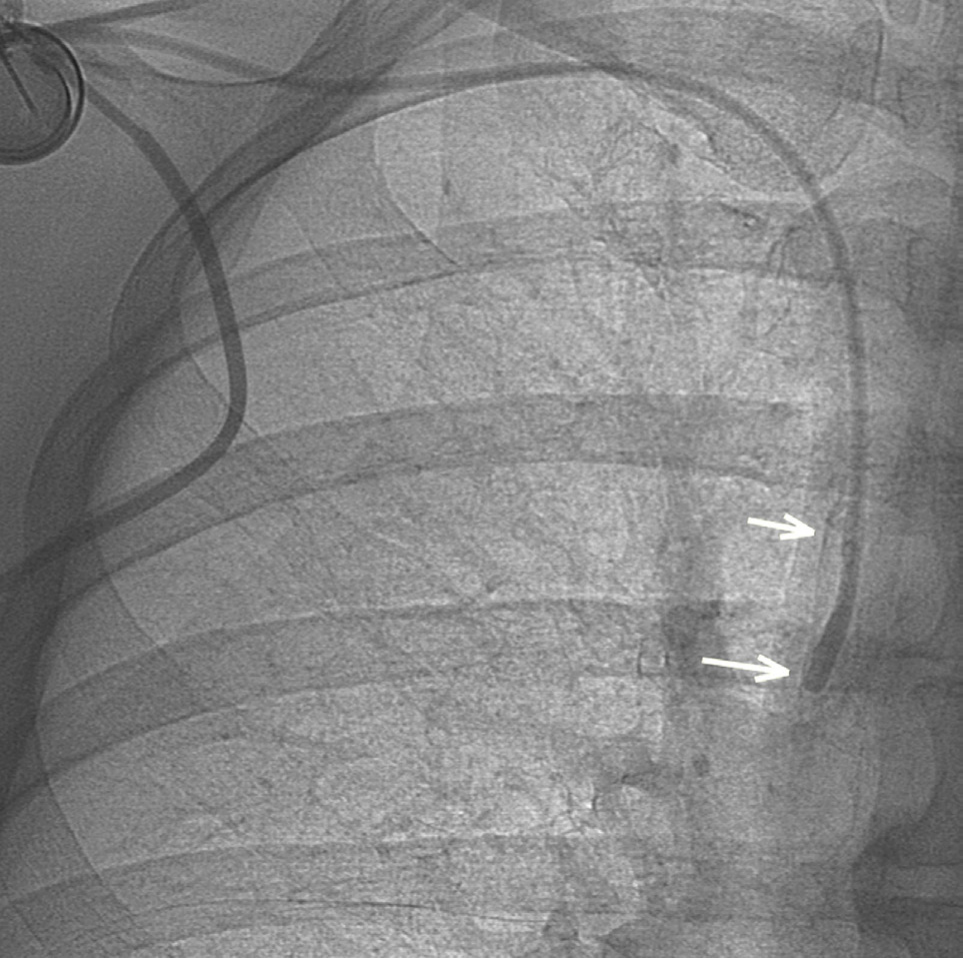
**After contrast media injection, a small pouch of contrast is visible at the tip of the catheter as long as a retrograde contrast tracking along the catheter (arrows), indicating fibrin sheath formation**.

A 41-year-old woman, with stage 4 mucinous breast carcinoma being treated with chemotherapy for 2 months, was referred to the Radiology Department for IVAD dysfunction. We evaluated the catheter by injecting a contrast media *via* the injection port under fluoroscopy, which revealed reflux with a small pouch of opacification around the catheter tip suggestive of fibrin formation (Figure 1).

**Figure 2:**
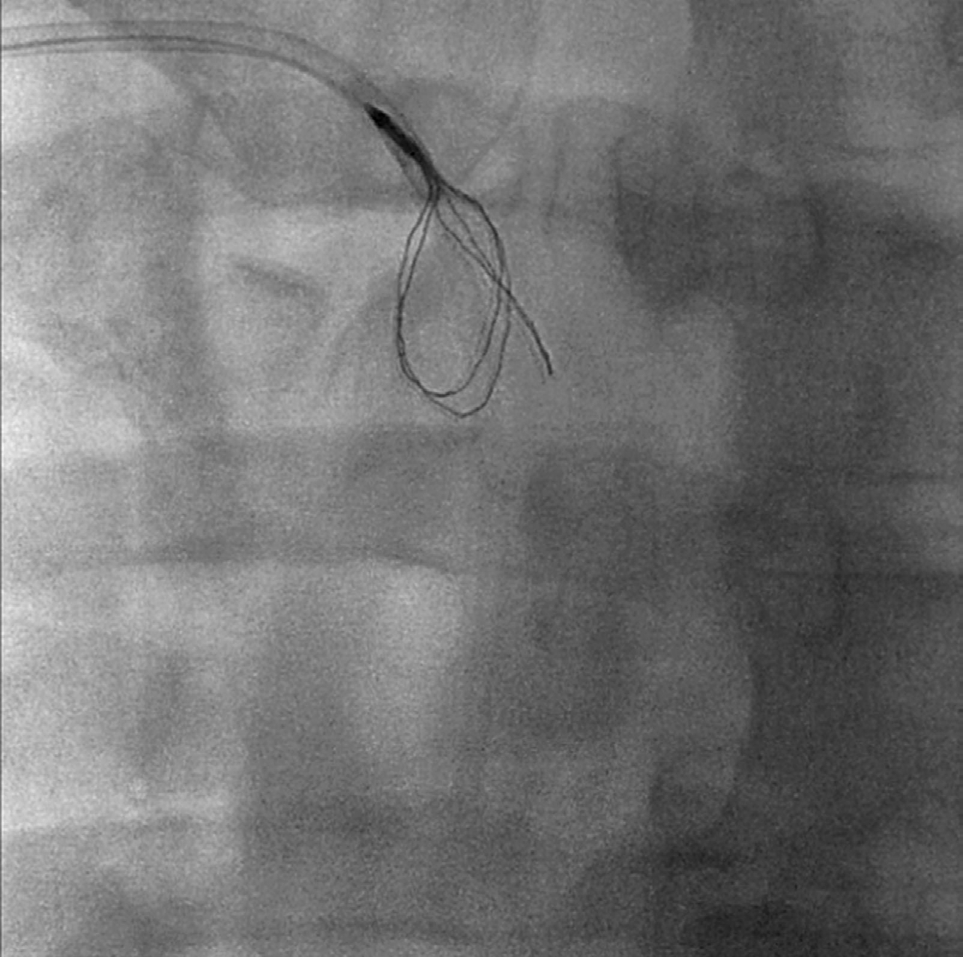
**In order to remove the fibrin sheath, the Atrieve Snare was inserted through a Judkins-Left 4 catheter and deployed just distally to the tip**.

**Figure 3:**
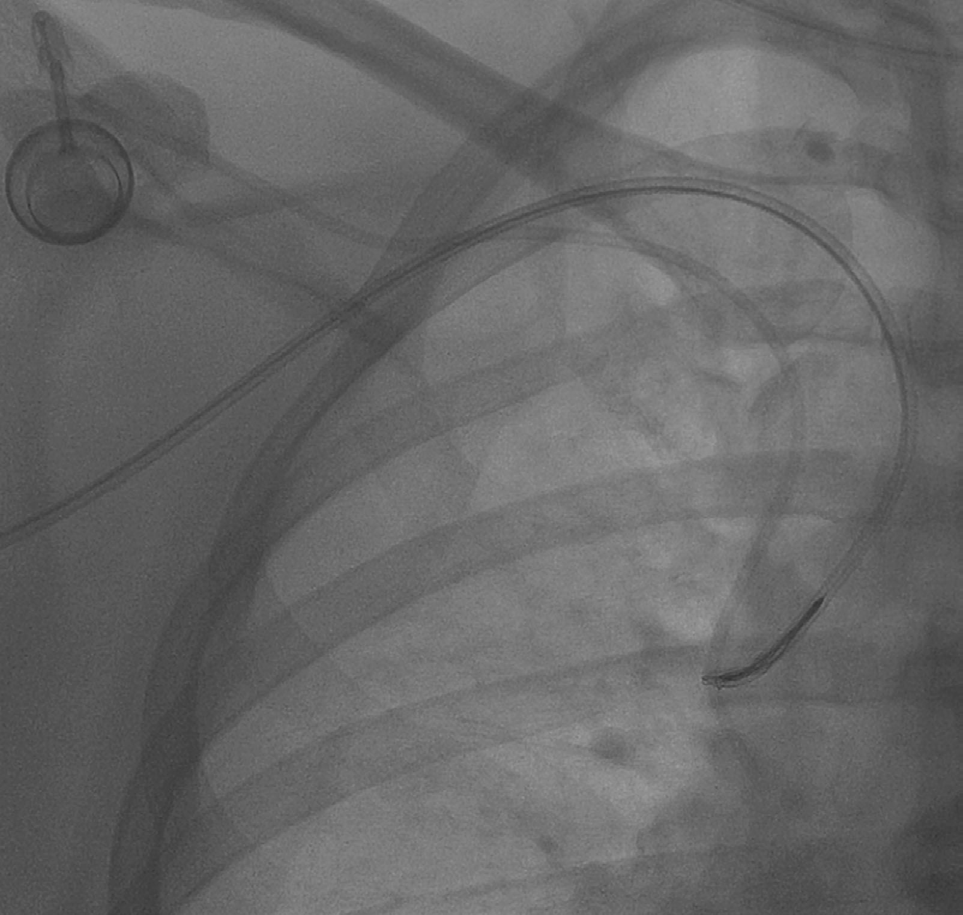
**Then, the snare was retrieved so as to encircle the catheter, and then it was loosely tightened proximally**.

**Figure 4:**
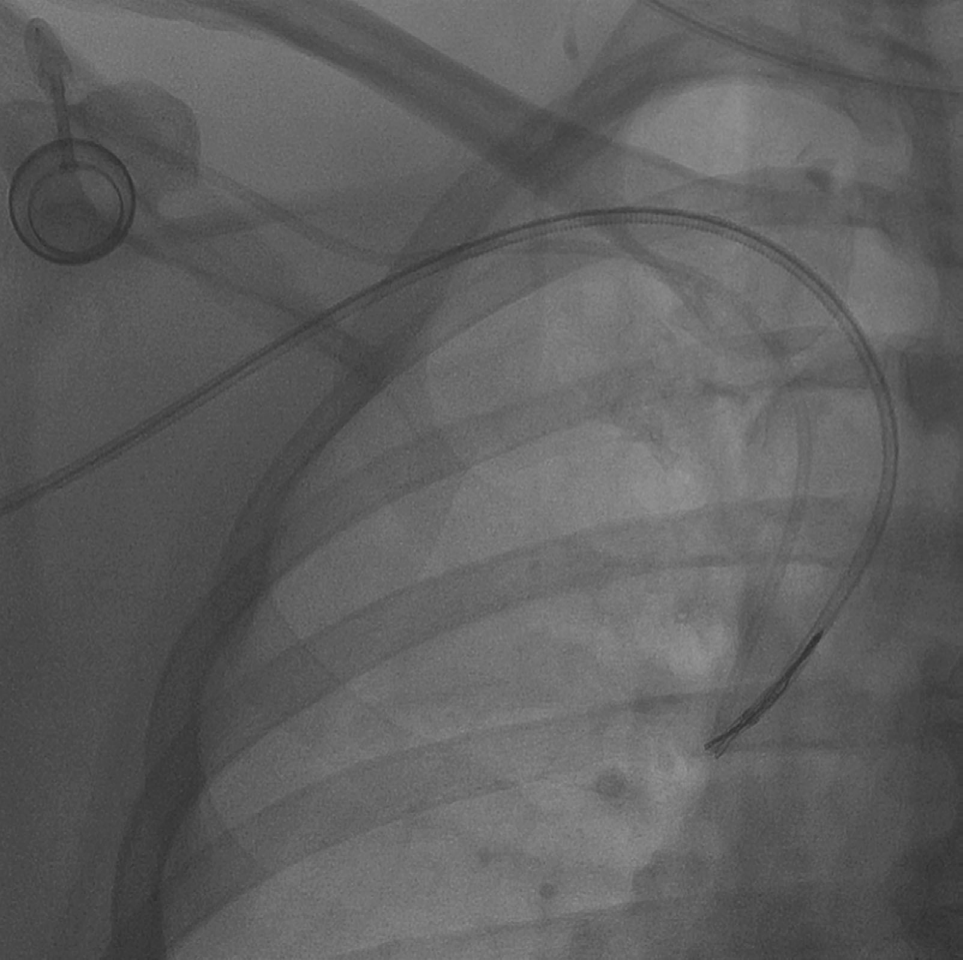
**The snare was kept tightened, and then it was pushed toward catheter’s tip in order to release fibrin**. No port displacement is visible.

In order to facilitate early discharge of the patient, we decided to obtain central venous access *via* the right brachial vein. Informed consent was obtained from the patient, and the right arm was abducted and prepared in sterile manner. Under local anesthesia, the right basilic vein was punctured using a micropuncture technique under ultrasound guidance. A 6 F × 45 cm introducer sheath (Terumo Europe, Leuven, Belgium) was then advanced on a hydrophilic 0.035″ × 150 cm guidewire (Terumo Europe) until its distal end reached the right axillary vein. A venogram was performed *via* the sheath excluding thrombus in the superior vena cava (SVC). A 5.2-F Judkins-Left 4 (JL4) catheter (Cordis Corporation, FL, USA) was subsequently positioned in the SVC distal to the tip of IVAD. An Atrieve™ Vascular Snare 15–9 mm (Angiotech, Medical Device Technologies Inc., Gainesville, FL, USA) was advanced inside the JL4 catheter. The snare was deployed and manipulated in order to encircle the tip of IVAD. The JL4 catheter and snare were then withdrawn proximally in the SVC while maintaining the snare coils open (Figure 2). After this manipulation, the snare was retracted into the JL4 catheter thus tightening the snare coils around the IVAD tip. The JL4 catheter and snare were pushed to remove fibrin. This is unlike the standard stripping technique *via* femoral approach where the snare is pulled back rather than pushed on the IVAD tip (Figures 3 and 4).

**Figure 5:**
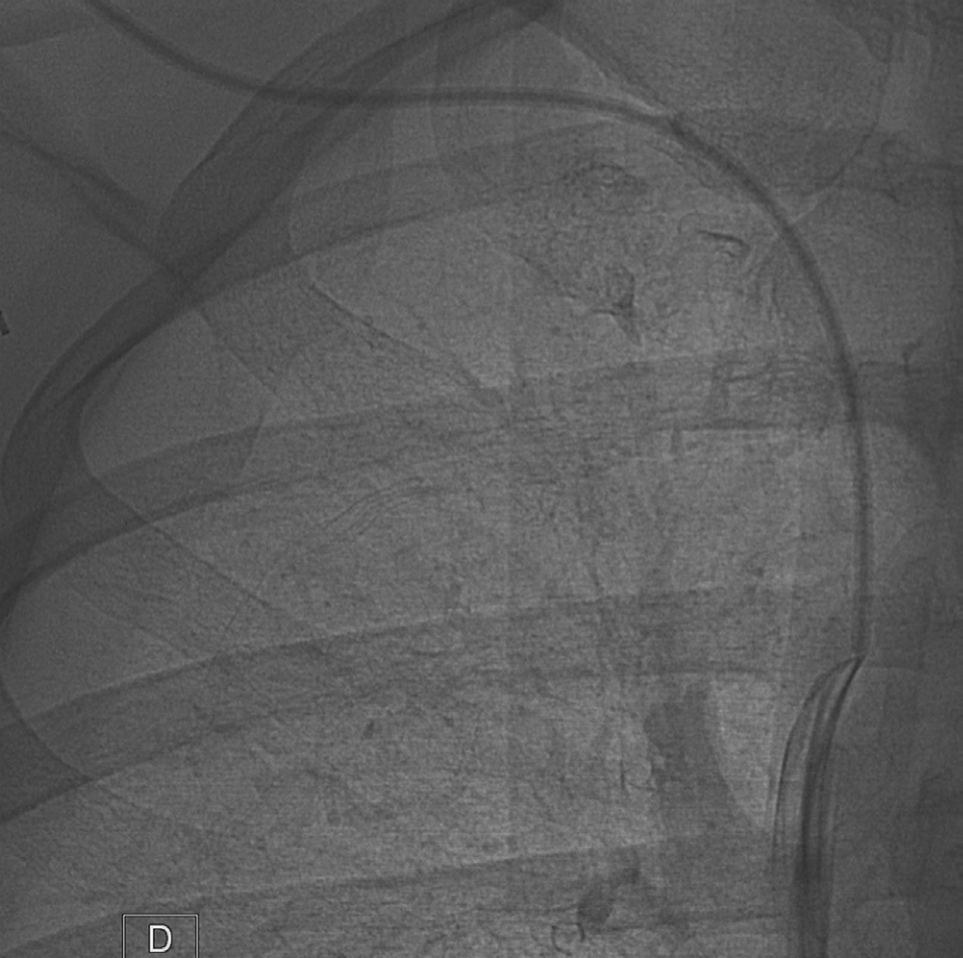
**Implanted venous access device opacification after fibrin sheath removal shows normal flow at the catheter tip**.

After two stripping maneuvers, injection of contrast media *via* the IVAD injection port did not demonstrate any reflux along the device catheter (Figure 5), and hence, adequate blood flow *via* the IVAD was reestablished. The introducer sheath was removed, and manual compression was applied to the puncture site. The patient was discharged 30 min after the procedure. No post-procedural complications were observed. The IVAD remained patent at 2-year follow-up.

## Discussion

Fibrin sheath removal is a well-described endovascular method to restore patency of IVAD (3). A long catheter is advanced, *via* femoral venous access, to the SVC, and the intervention is performed using a snare that encircles and captures the catheter.

Occasionally, femoral access is not technically feasible or contraindicated, and in some cases, arm access is preferred. An alternative venous access is preferred when faced with a thrombosis of the femoral vein, iliac vein or IVC and in geriatric patients with incontinence (4). Additionally, bilateral groin infections, prior surgery in the inguinal regions or previous inguinal or pelvic radiation therapy contraindicate femoral vein catheterization (5). Additionally, congenital anomalies of the IVC, such as interruption of IVC with azygos continuation, prevent fibrin sheath removal using standard femoral vein access.

In certain cases, the interventional radiologist may fail to perform stripping *via* femoral vein access. An unfavorable catheter orientation in a wide SVC or a catheter attached to the vessel wall may prevent the snare from encircling the catheter. However, access through the arm can aid the radiologist to surmount this challenge by providing a more favorable access route.

There are some advantages of brachial vein approach when compared to the femoral vein approach. Femoral vein catheterization has a risk of ecchymosis and local hematoma after sheath retrieval especially in anticoagulated patients or in patients with coagulopathies (6). Excellent hemostasis can be achieved using the brachial vein access because of their superficial location.

Moreover, when interventional radiologists opt for basilic vein access, erroneous puncture of the adjacent artery is avoided (7) because the basilic vein is typically situated at a safe distance from brachial artery.

In addition, bed rest is not necessary following brachial access interventions, allowing early discharge immediately after sheath removal (6, 7). Conversely, in most cases, concerning femoral access intervention, bed rest, and 4–6 h of observation are required. Brachial access allows for immediate patient discharge after the intervention avoiding post-procedural nursing and consequently reduces costs.

One disadvantage of brachial venous access is that the caliber of the arm veins is smaller than that of femoral veins. Consequently, catheterization could be more challenging thus increasing the risk of thrombosis. The diameter of the selected vein must be larger than the size of the introducer sheath. A 6-F introducer sheath, for example, requires that the vein diameter to be greater than or equal to 2 mm. An ultrasound of both arms before stripping would allow the interventional radiologist to select the larger vein. Preferably, the radiologist may catheterize the basilic vein as it has some advantages compared to others arm veins: it is larger, more superficial, and has no adjacent artery (7).

Another challenge concerning stripping *via* the brachial access is that the maneuver is performed by pushing the snare toward the distal portion of the IVAD tip. This can be more technically challenging than the retrieving maneuver during stripping *via* the femoral access. Additionally, attention must be given to catheter selection through which the snare is inserted. As in our case, where an ipsilateral venous access was selected, catheters such as JL4 can be selected in order to reach and encircle the catheter tip. Moreover, the radiologist must study the position and orientation of the device catheter in the SVC and choose the most favorable venous access and appropriate catheter in order to perform the intervention with safety and rapidity.

Complications concerning brachial vein puncture are rare. Engmann and Asch (7) studied a group of 74 patients where a Simon Nitinol^®^ filter was placed *via* the antecubital vein. They noted, as a minor complication, one occurrence of inadvertent brachial artery puncture during an attempt to access the brachial vein, with a formation of a small local hematoma. No patients presented with symptomatic access-site thrombosis. In another study, comparing antecubital vs. femoral venous access for right heart catheterization, Roule et al. (8) found fewer access-site hematomas after antecubital vein puncture.

One complication related to fibrin sheath removal is distal embolization of the fibrin to the pulmonary circulation, which is rarely symptomatic. No significant difference in embolization frequency is expected between the femoral and brachial techniques.

An alternative to brachial approach could be the jugular vein. The right jugular vein has direct access to the SVC. Similar to the brachial approach, fibrin sheath removal could be performed by the pushing maneuver. However, the jugular venous puncture is reported to be associated with specific complications (e.g., pneumothorax, air embolism, and carotid injury) that could not be observed in the brachial puncture. Furthermore, the jugular access needs post-procedural care of at least few hours that is not required with the ambulatory brachial approach.

## Conclusion

A case of IVAD stripping *via* brachial venous access is described. The step-by-step procedure does not differ significantly compared to IVAD stripping *via* femoral access but may be more technically challenging. This method could be used when femoral access is contraindicated or not technically feasible. Moreover, patients can benefit from earlier discharge and shorter hospital monitoring. Further evaluation is necessary in order to prove the feasibility and effectiveness of this technique.

## Ethics Statement

Written informed consent was obtained from the participant for the publication of this case report.

## Author Contributions

All authors listed have made substantial, direct, and intellectual contribution to the work and approved it for publication.

## Conflict of Interest Statement

The authors declare that the research was conducted in the absence of any commercial or financial relationships that could be construed as a potential conflict of interest.
